# Essential Oils of *Thymus* Species Against *Phytophthora* Species: A Structured Review and Novel In Vitro Evaluations

**DOI:** 10.3390/pathogens15060582

**Published:** 2026-05-28

**Authors:** Chiara Antonelli, Najwa Benfradj, Anna Maria Vettraino

**Affiliations:** Department for Innovation in Biological, Agro-Food and Forest Systems (DIBAF), University of Tuscia, 01100 Viterbo, Italy

**Keywords:** essential oil, biological control, sustainable disease management, biofungicides, oomycetes

## Abstract

*Phytophthora* species are among the most destructive plant pathogens worldwide, causing severe losses in agricultural, forest, and natural ecosystems. In recent years, the management of *Phytophthora* diseases has increasingly shifted toward eco-sustainable strategies, with growing interest in plant-derived extracts, particularly essential oils, as low-risk alternatives to synthetic fungicides. In this study, a structured review was combined with new in vitro assays to assess the antifungal activity of essential oils from *Thymus vulgaris* (TV-EO) and *T. serpyllum* (TS-EO) against *P. cinnamomi, P. drechsleri*, *P. cactorum*, *P. citrophthora*, *P. nicotianae*, *P. palmivora*, and *P. infestans*. Literature searches were conducted in April 2025 using the Web of Science and Scopus databases, following PRISMA guidelines, with the search term “*Thymus*” or “Thyme” and “*Phytophthora*”. Twenty studies included in the review demonstrated that the activity of *Thymus* essential oils against *Phytophthora* species was highly variable and shaped by chemotype, *Thymus* species, pathogen, and experimental setup. Additional in vitro assays further confirmed a clear dose-dependent inhibitory effect for both TV-EO and TS-EO. TS-EO consistently exhibited stronger activity than TV-EO, likely reflecting its carvacrol-rich chemotype, while thymol-based TV-EO showed lower but still significant inhibition depending on the pathogen species. Overall, these results highlight the potential of *Thymus* essential oils as eco-friendly tools for the management of *Phytophthora* diseases. However, the strong dependence on chemotype, pathogen species, and assay conditions underscores the need for standardized testing, detailed chemical characterization, and in vivo validation.

## 1. Introduction

Several species of the genus *Phytophthora* are among the most destructive plant pathogens worldwide, threatening a wide range of agricultural, forestry, ornamental and natural ecosystems and causing substantial economic, environmental, and social losses [1,2,3,4]. Although not exhaustive, the following examples illustrate the extensive impact of *Phytophthora* diseases worldwide. *Phytophthora infestans* was the causal agent of the Irish potato famine [5] and currently causes annual economic losses exceeding 6 billion USD due to yield reductions and management costs [6]. *Phytophthora nicotianae* is common in nurseries and urban green spaces, where it can dominate local *Phytophthora* populations [7,8]. For example, in Athens, Greece, it accounted for 85% of isolates recovered from symptomatic plane trees (*Platanus orientalis* and *P. x acerifolia*); a total of 41% of infected trees died within five years, underscoring its significant ecological and economic impact [9]. *Phytophthora* species are particularly difficult to control due to their broad host range and ability to spread through soil, water, infected plant material, and contaminated tools, which facilitates their rapid dissemination in both nurseries and field conditions [10]. Interspecific hybridization and the emergence of new genotypes have further contributed to increased virulence and to the expansion of the host range [11,12,13]. Moreover, the reproductive strategies of *Phytophthora* species, together with their ability to form long-lived dormant structures, enhance their survival under unfavourable environmental conditions and make their eradication challenging [10,14,15]. Together, these factors make management of *Phytophthora* infections difficult and highlight the need for alternative control strategies that are low-risk to human health and the environment [16,17,18,19].

Among potential eco-friendly alternatives, plant extracts are particularly relevant, as they are known for their antimicrobial activity [20,21,22]. In this context, essential oils (EOs) derived from *Thymus* species have been widely recognized for their strong efficacy against a broad range of plant-pathogenic fungi and oomycetes, including *Alternaria alternata*, *Trametes versicolor*, *Pleurotus ostreatus*, *Poria monticola*, and *Gloeophyllum trabeum* [23,24,25]. However, despite their recognized anti-microbial potential, the efficacy of *Thymus* EOs against *Phytophthora* species, belonging to the class Oomycetes, remains insufficiently investigated.

Therefore, this study aimed to clarify the current state of knowledge on the anti-oomycetes activity of *Thymus* EOs against *Phytophthora* species by conducting a literature review. In parallel, new in vitro assays were conducted to address existing knowledge gaps and to support the development of eco-sustainable disease-management strategies.

## 2. Materials and Methods

### 2.1. Literature Search Strategy

The analysis was carried out following the PRISMA (Preferred Reporting Items for Systematic Reviews and Meta-Analysis) guidelines [26] (Table S1). Scientific papers examining the efficacy of EOs derived from *Thymus* species against *Phytophthora* species were retrieved through a systematic search of the Web of Science (WoS, accessed on 4 April 2025) and Scopus (accessed on 4 April 2025) databases. The keywords used were: “*Thymus*” or “Thyme” and “*Phytophthora*”. Papers met the following inclusion criteria: original studies investigating the efficacy of EOs extracted from *Thymus* species against *Phytophthora* species.

The following records were excluded from the analysis: theses, proceedings, conference papers, reviews, studies not relevant to the scope of the review, non-English publications and studies that did not include in vitro assays.

The titles and abstracts of all records identified through the search were independently screened by two reviewers (C.A. and A.M.V.). Papers considered potentially eligible were subsequently evaluated to determine whether they met the established inclusion and exclusion criteria.

From all eligible studies, two authors independently extracted the following information: *Thymus* species, *Phytophthora* species, antimicrobial method and experimental conditions applied. No formal review protocol was prepared, and the study was not registered in a review database.

### 2.2. Novel EOs–Pathogen Interactions

#### 2.2.1. *Phytophthora* Isolates

Isolates of *Phytophthora cinnamomi* Ph28, *P. nicotianae* Tu2.1, *P. drechsleri* P11, *P. cactorum* P69, *P. citrophthora* P6, *P. palmivora* P1 and *P. infestans* AMV23 were retrieved from the culture collection of Anna Maria Vettraino at PhytoLab+ (Phyto Innovation Lab—DIBAF, University of Tuscia, Viterbo, Italy).

#### 2.2.2. *Thymus* EOs

The EOs of *Thymus vulgaris* (TV-EO) and *T. serpyllum* (TS-EO) were purchased from Flora srl (Florence, Italy) and previously characterized by GC-MS [25,27]. The relative proportions of the major compounds present in each tested EO are reported in Table 1.

#### 2.2.3. In Vitro Efficacy of *Thymus* EOs Against *Phytophthora* Species

Before use in the assays, the EOs were emulsified using Tween 80 (0.5%, *v*/*v* final concentration) to obtain homogeneous dispersions. Emulsions were incorporated at different concentrations (0, 100, 200, 300, and 500 ppm) into Potato Dextrose Agar (PDA, 39 g L^−1^; VWR International, Milan, Italy) for *P. cinnamomi* Ph28, *P. nicotianae* Tu2.1, *P. drechsleri* P11, *P. cactorum* P69, *P. citrophthora* P6 and *P. palmivora* P1 or in Pea Agar (PEA, 120 g L^−1^ peas, 15 g L^−1^ agar; VWR International, Milan, Italy) for *P. infestans* AMV23, immediately before pouring the media into Petri dishes, at a temperature of 45 °C. Controls included untreated media and media containing 0.5% Tween 80. Mycelial plugs (5 mm) from 7-day-old cultures of each *Phytophthora* isolate were transferred to fresh PDA/PEA, either amended with EOs or controls. Cultures were incubated in the dark at 25 °C for *P. cinnamomi* Ph28, *P. nicotianae* Tu2.1, *P. drechsleri* P11, *P. cactorum* P69, *P. citrophthora* P6, and *P. palmivora* P1, and at 18 °C for *P. infestans* AMV23, for a period of seven days. Each treatment included five replicates. For *P. infestans* AMV23, complete inhibition was observed at 100 ppm, and the assay was repeated using lower concentrations (12.5, 25, 37.5, and 50 ppm). The antimicrobial efficacy was evaluated using Mycelial Growth Inhibition (MGI, %):

MGI = (C − T)/C × 100,

where “C” is the mean value of the colony diameter (mm) of controls and “T” is the mean colony diameter of the treated cultures.

For each *Phytophthora* species, the value of the Minimum Inhibitory Concentration (MIC) was assessed as the lowest EO concentration that completely inhibited the pathogens’ growth. To determine whether the treatments were fungicidal or fungistatic, at the end of the experiment, mycelial plugs were transferred to fresh PDA/PEA plates and incubated as described above. Absence of mycelial growth indicated a fungicidal effect, whereas resumption of mycelial growth indicated a fungistatic effect.

#### 2.2.4. Statistical Analysis

Statistical analyses were performed separately for *P. infestans* and for the remaining *Phytophthora* species, because *P. infestans* was tested at lower concentrations than the other species due to its higher sensitivity to the EOs. The normal distribution of the data was evaluated by assessing model residuals with the Shapiro–Wilk test. The experimental run, corresponding to the two independent repetitions of the experiment, Exp1 and Exp2, was included in the models as a control factor to account for possible variability between experimental runs. This factor was distinct from the replicate factor, which referred to replicate plates within each treatment.

For *P. infestans*, percentage inhibition data were analyzed using a two-way ANOVA, while for the remaining *Phytophthora* species, data were analyzed using a multifactorial ANOVA. Effect size was estimated using partial eta squared, η^2^p. Post hoc comparisons among estimated marginal means were performed with Tukey correction for multiple comparisons. Dose effects were evaluated within each species–EO combination, EO effects within each species–dose combination, and species effects within each EO–dose combination, excluding *P. infestans*. All statistical analyses were performed in R software version 4.3.2. [28], using the packages emmeans, multcomp, car, effectsize, dplyr, and openxlsx.

## 3. Results

### 3.1. Literature Review

**Figure 1:**
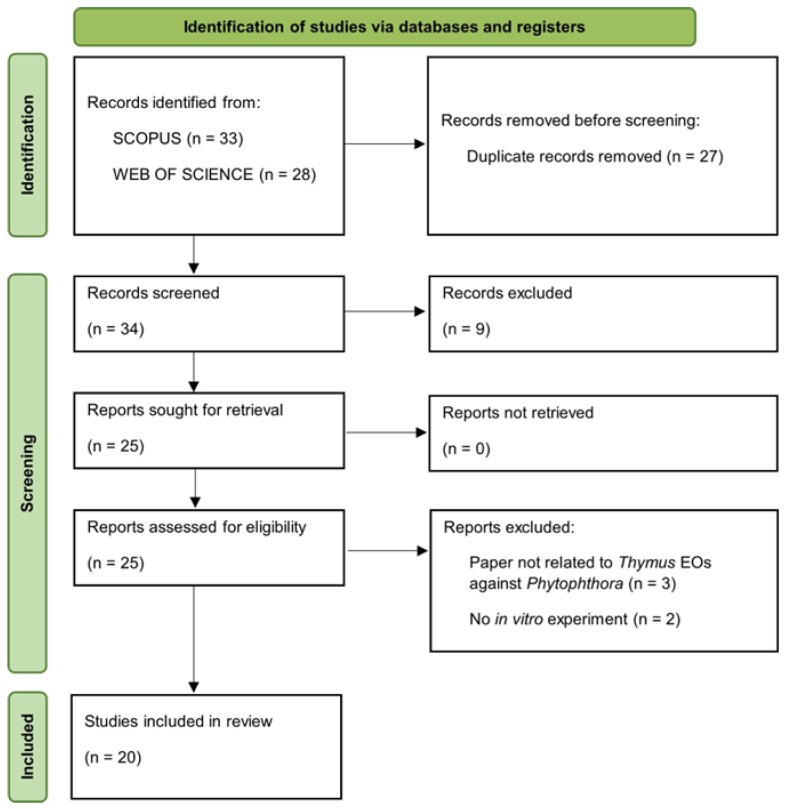
PRISMA flowchart illustrating the experimental study search and selection process.

Twenty original papers published between 2002 and 2025 reporting the efficacy of *Thymus* EOs against *Phytophthora* species in in vitro tests were included in this study (Figure 1).

**Figure 2:**
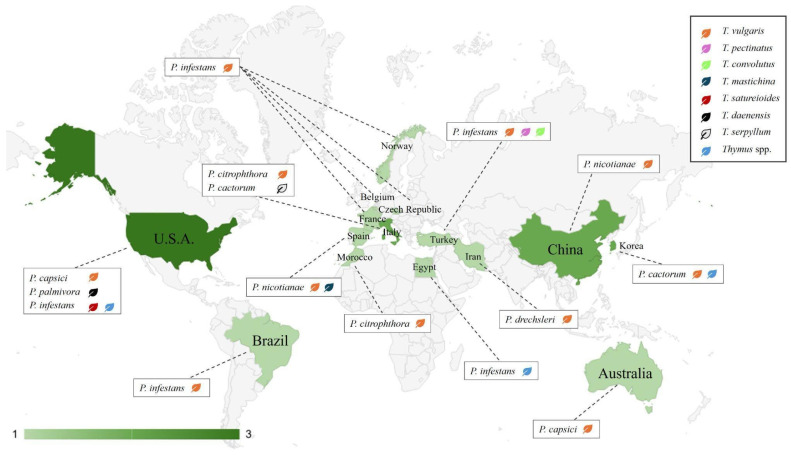
Geographical distribution of the studies included in the review, showing the countries where in vitro assays of *Thymus* EOs against *Phytophthora* species were conducted. The intensity of the country colour indicates scientific productivity (1–3 papers), whereas different *Thymus* species are represented by leaf symbols of different colours.

The studies were conducted in 14 different countries (Figure 2).

**Figure 3:**
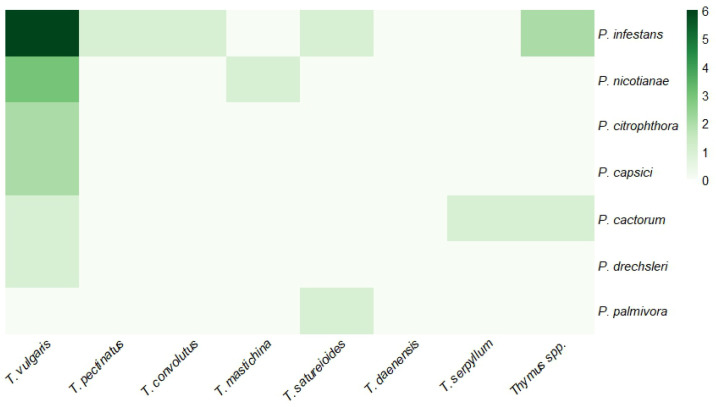
Heatmap of the *Thymus*–*Phytophthora* species combinations identified across the included studies. The green colour gradient, ranging from light to dark, reflects the increasing number of studies reported for each species combination.

Overall, the efficacy of EOs from a total of seven *Thymus* species (*T. vulgaris*, *T. pectinatus*, *T. convolutus*, *T. mastichina*, *T. satureioides*, *T. daenensis*, *T. serpyllum*) has been investigated against seven *Phytophthora* species (*P. infestans*, *P. cactorum*, *P. drechsleri*, *P. citrophthora*, *P. palmivora*, *P. nicotianae*, *P. capsici*). Among the species considered, *P. infestans* and *T. vulgaris* were the most extensively studied, being investigated in approximately 40% and 75% of the selected publications, respectively (Figure 3, Table 2).

Only 11 of the 20 selected papers reported the chemical characterization of the *Thymus* EOs used. Among these studies, thymol was the predominant compound in 57% of the oils analyzed, mainly in *T. vulgaris* and *T. daenensis*, followed by eucalyptol in 21% of cases, detected in *T. convolutus*, *T. mastichina*, and *T. vulgaris*, and carvacrol in 14%, found in *T. serpyllum* and *T. vulgaris*. In contrast, Camele et al. [29] evaluated the biocontrol efficacy of *T. vulgaris* EOs characterized by a high abundance of *o*-cymene (56.20%) (Table 2).

**Table 2:** Overview of in vitro assays evaluating the activity of *Thymus* essential EOs against *Phytophthora* species.

*Phytophthora* Species	*Thymus* Species	Main Compound	Methods	Results	References
*P. infestans*	*T. vulgaris*	thymol (n.d.)	MGI ZPI FMGI	100% = 500 μL L^−1^ 100% = 750 μL L^−1^ 100% = 500 μL L^−1^	Horst et al. [30]
	*T. vulgaris*	thymol (51.00%)	SGI (microplate)	IC50 = 99.41 mg L^−1^	Deweer et al. [31]
	*T. vulgaris* *T. pectinatus* *T. convolutus*	carvacrol (72.40%) thymol (52.82%) eucalyptol (24.47%)	FMGI	100% = 1 μL/disc 100% = 2 μL/disc 60% = 4 μL/disc	Aksit et al. [23]
	*T. vulgaris*	-	MGI	100% = 0.25–0.4 μL mL^−1 (1)^	Mazáková et al. [32]
	*T. vulgaris*	-	MGI SGI (microplate)	100% = 0.83 μL mL^−1^ ED50 = 0.12 μL mL^−1^	Najdabbasi et al. [33]
	*Thymus* sp.	-	MGI	100% = 8 mL L^−1^	El-Mohamedy and Abd-El-Latif [34]
	*Thymus* sp. *T. satureioides*	-	MGI	50.3–94.4% = 100 ppm ^(2)^ 87.1–99.4% = 100 ppm ^(2)^	Olanya and Larkin [35]
	*T. vulgaris*	thymol (52.71%)	1 screening: well diffusion 2 screening: MGI	89% = 4 uL/well 100% = 1:30 000	Quintanilla et al. [36]
*P. cactorum*	*T. serpyllum*	carvacrol (68.49%)	MGI	100% = 480 μg mL^−1^ EC50 = 120.453 μg mL^−1^	Vettraino et al. [27]
	*Thymus* species	-	FMGI (*Thymus* alone) FMGI (*Thymus* + Origanum)	<20% = 1 μL/disc T-EO 100% = 2.5 μL/disc T-EO + 1 μL/disc O-EO	Park et al. [37]
	*T. vulgaris*	thymol (47.36%)	FMGI	100% = 14 × 10^−3^ mg mL^−1^ air	Kim et al. [38]
*P. nicotianae*	*T. vulgaris*	thymol (39.77%)	MGI ZGI	100% = 240 μg mL^−1^ 100% = 80 μg mL^−1^	Lu et al. [39]
	*T. vulgaris*	thymol (39.77%)	MGI SPI ZPI ZGI	100% = 216 mg L^−1^ 100% = 72 mg L^−1^ 100% = 72 mg L^−1^ 100% = 72 mg L^−1^	Lu et al. [40]
	*T. vulgaris* *T. mastichina*	eucalyptol (37.48%) eucalyptol (43.26%)	disc diffusion	75% = 30% (*v*/*v*); ED50 = 13.1% (*v*/*v*) 79% = 30% (*v*/*v*); ED50 = 22% (*v*/*v*)	Diánez et al. [41]
*P. palmivora*	*T. daenensis*	thymol (73.30%)	MGI	100% = 200 μL L^−1^	Sarkhosh et al. [42]
*P. citrophthora*	*T. vulgaris*	-	well diffusion	73% = 50 μL/well	Nassiri et al. [43]
	*T. vulgaris*	o-cymene (56.20%)	MGI	≈100% = 250 ppm	Camele et al. [29]
*P. capsici*	*T. vulgaris*	-	MGI FMGI SPI ZPI SGI ZGI	EC50 = 0.14 μg mL^−1^ EC50 = 0.11 μg mL^−1^ EC50 = 0.03 μg mL^−1^ EC50 = 0.06 μg mL^−1^ EC50 = 0.08 μg mL^−1^ EC50 = 0.09 μg mL^−1^	Bi et al. [44]
	*T. vulgaris*	-	MGI (EO blend (EOB) of citronella, red *Thymus*, and clove bud)	75.12% = 125-fold diluted EOB	Wise and Selby-Pham [45]
*P. drechsleri*	*T. vulgaris*	-	MGI	IC50 = 0.147% (*v*/*v*); MIC = 0.4% (*v*/*v*); MFC= 0.8% (*v*/*v*)	Mohammadi et al. [46]

^(1)^ Range based on 20 isolates; ^(2)^ Range based on 4 different genotypes; MGI: Mycelial Growth Inhibition (%); ZPI: Zoospore Production Inhibition (%); FMGI: Mycelial Growth Inhibition by fumigation (%); SGI: Sporangia Germination Inhibition (%); ZGI: Zoospore Germination Inhibition (%); SPI: Sporangia Production Inhibition (%).

The available studies consistently indicate that *Thymus* EOs exert strong inhibitory activity against multiple *Phytophthora* species. However, the strength of evidence varies markedly depending on experimental design, biological replication, and chemical characterization [29,30,31,32,33,34,35,36,37,38,39,40,41,42,43,44,45,46]. Across studies, efficacy is frequently reported as complete or near-complete inhibition of mycelial growth, zoospore development, or sporangial processes, particularly for *P. infestans*, *P. cactorum*, and *P. nicotianae*. This consistency across species and assays supports a broad-spectrum anti-oomycete potential of *Thymus*-derived volatiles.

Despite this overall convergence, the interpretability of results is strongly constrained by methodological heterogeneity. A dominant limitation is the reliance on single-isolate experimental systems, which restricts inference about population-level sensitivity and overlooks known intra-species variability in *Phytophthora*. Studies incorporating multiple isolates or genotypes consistently provide more conservative but ecologically realistic estimates of efficacy, highlighting that isolate diversity is a critical determinant of robustness [32,35,44].

A second major axis of variation relates to experimental rigour and reproducibility. Studies that include independent experimental repetitions, appropriate positive controls (e.g., commercial fungicides), and dose–response modelling (IC_50_/EC_50_ estimation) provide substantially stronger evidence than single-experiment screening assays. In contrast, reports lacking independent replication or statistical depth tend to overestimate efficacy and limit comparability across experiments [31,33,34,39,46].

Chemical characterization of EOs emerges as another key determinant of interpretability. Only a subset of studies reports GC–MS profiles, yet these consistently show thymol, carvacrol, and eucalyptol as dominant constituents. Where chemical data are available, antifungal activity is more readily contextualized within chemotype variation, reinforcing the concept that bioactivity is chemotype-dependent rather than strictly species-dependent [23,27,29,30,31]. The absence of chemical profiling in several studies therefore represents a major gap, preventing meaningful cross-study synthesis.

Methodologically, studies employing multiple complementary endpoints (mycelial growth, zoospore inhibition, sporangial production, and fumigation assays) and mechanistic investigations (e.g., membrane disruption, microscopy) provide the most comprehensive insight into modes of action [30,33,40,44]. These studies suggest that *Thymus* oils do not act solely on vegetative growth but can disrupt multiple developmental stages of the pathogen, reinforcing their potential as multi-target biofungicides.

Importantly, studies integrating formulation approaches (e.g., nanoencapsulation or emulsified systems) consistently report improved stability and activity, underscoring that volatility and physicochemical constraints are central to the performance of EOs [27]. This indicates that efficacy under laboratory conditions may underestimate potential performance in optimized delivery systems.

Overall, the evidence supports a coherent conclusion: *Thymus* EOs represent a promising source of anti-*Phytophthora* compounds with multi-stage inhibitory activity. However, the current literature is heavily weighted toward simplified in vitro screening systems with limited biological replication and incomplete chemical standardization [29,30,31,32,33,34,35,36,37,38,39,40,41,42,43,44,45,46]. Consequently, while the direction of effect is consistent, the magnitude of efficacy remains context-dependent and not yet fully generalizable.

Future studies should therefore prioritize multi-isolate experimental designs, standardized reporting of independent experimental runs, full GC–MS characterization, and harmonized dose–response frameworks to enable cross-study comparability and meta-analytical synthesis.

### 3.2. Novel EOs–Pathogen Interactions

**Figure 4:**
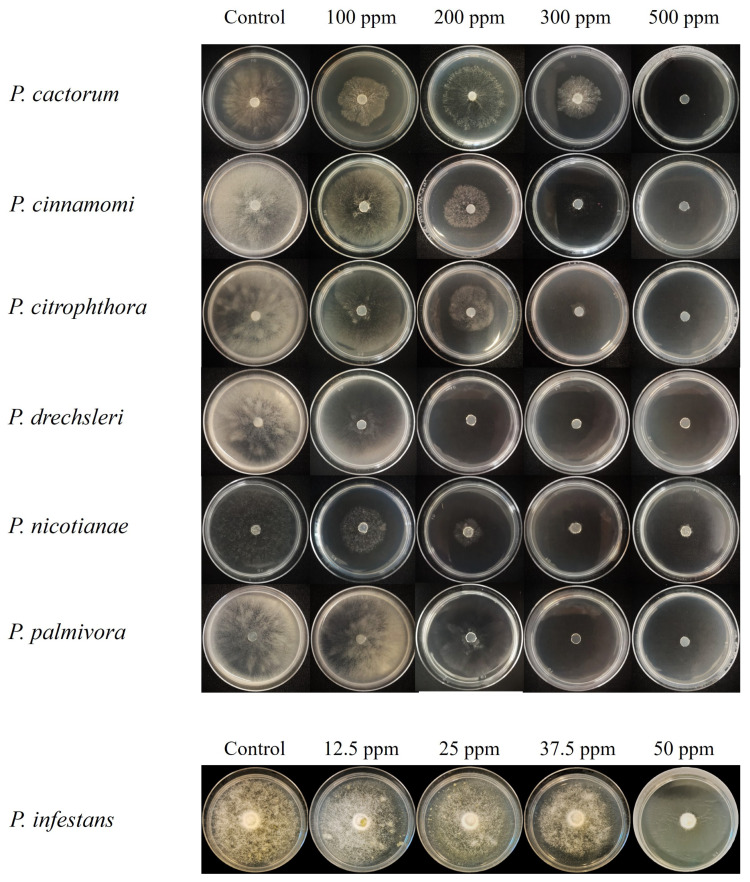
Mycelial growth of *Phytophthora* species at different concentrations of TV-EO.

**Figure 5:**
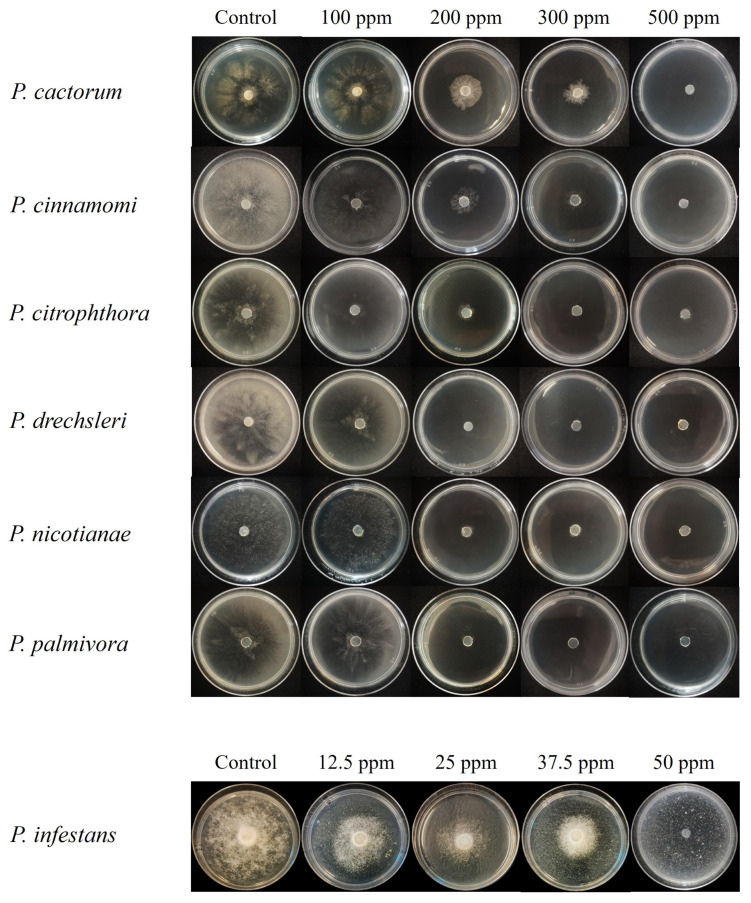
Mycelial growth of *Phytophthora* species at different concentrations of TS-EO.

The inhibitory activity of TV-EO and TS-EO against the tested *Phytophthora* species is summarized in Table 3, while representative images of the in vitro plate assays are shown in Figure 4 and Figure 5. Percentage inhibition was not significantly affected by the experimental run in either *P. infestans* (F = 0.38, *p* > 0.05, ηp^2^ = 0.003) or the other *Phytophthora* species (F = 0.90, *p* > 0.05, ηp^2^ = 0.002). These results confirmed good reproducibility across experiments and supported the robustness of the assay. For *P. infestans*, ANOVA on percentage inhibition revealed a highly significant effect of dose, F = 227.82, *p* < 0.001, ηp^2^ = 0.939, and oil type, F = 49.01, *p* < 0.001, ηp^2^ = 0.454, as well as a significant oil × dose interaction, F = 3.84, *p* < 0.05, ηp^2^ = 0.206. A clear dose-dependent increase in inhibition was observed, with TS-EO generally showing higher inhibitory activity than TV-EO, particularly at the highest dose, where TS-EO reached complete inhibition.

For *P. palmivora*, *P. nicotianae*, *P. cactorum*, *P. cinnamomi*, *P. drechsleri* and *P. citrophthora*, overall ANOVA on percentage inhibition revealed a highly significant effect of dose, F = 2914.90, *p* < 0.001, ηp^2^ = 0.953; species, F = 42.78, *p* < 0.001, ηp^2^ = 0.313; and oil type, F = 6.81, *p* < 0.001, ηp^2^ = 0.117. Significant interactions were also detected for species × oil, species × dose, oil × dose, and species × oil × dose, indicating that the inhibitory response depended on both the pathogen species and the oil–dose combination. Species-specific analyses confirmed a strong dose-dependent increase in inhibition for all 6 tested *Phytophthora* species, with dose showing the largest effect size in every case. Oil type significantly affected inhibition in all species, while the oil × dose interaction was significant for *P. palmivora*, *P. nicotianae*, *P. cactorum*, and *P. citrophthora*, but not for *P. cinnamomi*. Post hoc comparisons showed that TS-EO generally produced higher inhibition than TV-EO at intermediate doses, particularly at 200 ppm, whereas both oils reached very high or complete inhibition at the highest dose.

The MIC and MFC values varied among the tested *Phytophthora* species, indicating species-specific sensitivity to the two *Thymus* EOs (Table 4). TS-EO generally showed stronger inhibitory activity than TV-EO, as reflected by lower MIC values in some species. TS-EO was highly effective against *P. infestans* AM23, showing both MIC and MFC values of 50 ppm. For *P. citrophthora* P6, TS-EO showed lower MIC and MFC than TV-EO, corresponding to 300 and 500 ppm, respectively. Both oils showed comparable activity against *P. drechsleri* P11 and *P. palmivora* P1, with MIC values of 200 and 300 ppm, respectively, and MFC values of 500 ppm. In contrast, no MIC or MFC was detected for TV-EO against *P. cactorum* P69, *P. nicotianae* Tu2.1, and *P. infestans* AM23 within the tested concentration range.

## 4. Discussion

Plants produce a wide range of secondary metabolites, including EOs, which have attracted increasing interest as natural alternatives to synthetic pesticides [22,47,48,49]. In the present study, a structured literature survey specifically highlighted the promising role of *Thymus* EOs in plant protection.

However, since the search was focused on selected keywords related to *Thymus* and *Phytophthora*, the use of broader or alternative search terms could retrieve additional studies and potentially modify the overall picture of the available literature. Despite this limitation, the studies included in the survey provide useful evidence supporting the inhibitory activity of *Thymus* EOs against several *Phytophthora* species.

Taken together, the reviewed studies consistently indicate that *Thymus* EOs possess anti-*Phytophthora* activity, although the strength and reproducibility of the reported effects depend strongly on the experimental design adopted. The most informative studies are those combining chemical characterization, appropriate controls, multiple independent experiments, dose–response analysis, and more than one pathogen isolate. Conversely, when these methodological details are not clearly reported, the results should be considered preliminary and interpreted with caution.

Direct comparison among studies remains difficult because assays differed substantially in EO composition, pathogen species and number of isolates, EO concentrations tested, exposure methods, and statistical approaches. The presence of methodological limitations should not be interpreted as meaning that these studies lack scientific value. Many of them provide useful preliminary evidence and contribute to identifying promising antifungal effects of *Thymus* EOs. However, the weight given to their conclusions should depend on the robustness of the experimental design. For example, studies based on a single isolate, limited replication, incomplete controls, or mainly qualitative observations can still be informative, but they are better considered as exploratory rather than definitive. Accordingly, the variability among studies reflects not only biological differences, but also important differences in methodological rigour and levels of evidence.

Despite the growing interest in plant-derived biofungicides, investigations on *Thymus* EOs against *Phytophthora* remain restricted to a relatively small number of pathogen and plant species combinations (Table 2). Most available studies focused on *P. infestans* and *T. vulgaris*, whereas other economically important *Phytophthora* species have received comparatively limited attention. Most studies focused on the control of *P. infestans*, demonstrating the high efficacy of *Thymus* EOs against this pathogen. Horst et al. [30] showed complete inhibition of mycelial growth and zoospore-related parameters using *T. vulgaris* EO, while Deweer et al. [31] provided a quantitative estimation of activity through IC50 values in a sealed microplate assay. Aksit et al. [23] also reported strong activity of different *Thymus* species, including *T. vulgaris*, *T. pectinatus*, and *T. convolutus*, allowing a partial link between chemical profile and biological activity. In addition, Mazáková et al. [32] adopted an approach that better accounted for variability among *P. infestans* isolates by including approximately twenty isolates, which represents an important strength compared with most other studies. Similarly, Olanya and Larkin [35] evaluated several *P. infestans* isolates representing different genotypes, showing that sensitivity to EOs may vary among pathogen populations.

Importantly, the reviewed literature suggests that *Thymus* EOs not only suppress vegetative mycelial growth but may also interfere with key reproductive stages of the pathogen life cycle, including sporangia and zoospore formation. This aspect is particularly relevant from an epidemiological perspective, since a reduction in zoospore production and germination may substantially affect pathogen dissemination. For example, Horst et al. [30] reported complete inhibition of *P. infestans* mycelial growth at 500 µL L^−1^ and suppression of zoospore release at 750 µL L^−1^, while Lu et al. [40] observed total inhibition of sporangia and zoospore formation at 72 mg L^−1^, compared to a higher MIC for mycelial growth (216 mg L^−1^). Collectively, these findings indicate that *Thymus* EOs may interfere with multiple stages of the oomycete life cycle, thereby increasing their potential relevance for integrated disease management strategies.

Another major factor influencing antifungal efficacy is the chemical composition of the EOs. In the reviewed studies, only part of the literature reported chemical characterization of the tested EOs, although this information is essential for reproducibility and comparison among studies. Thymol was frequently reported as a major compound in *T. vulgaris* and *T. daenensis* oils, whereas other chemotypes were characterized by compounds such as carvacrol, eucalyptol, or o-cymene [23,27,29,31,38,39,40,41]. Previous studies have emphasized that the antimicrobial activity of *Thymus* EOs may depend not only on the dominant compound but also on the overall chemical profile and possible synergistic interactions among major and minor constituents [25,50,51,52,53]. This variability is particularly important because EO composition is strongly influenced by species identity, chemotype, geographical origin, environmental conditions, harvest period and extraction procedure [50,51,52,53].

The novel in vitro assays performed in the present study are consistent with the general trends identified in the literature and further strengthen the evidence supporting the anti-*Phytophthora* potential of *Thymus* EOs. The lack of a significant experimental-run effect confirmed good reproducibility across the two independent experiments and supported the robustness of the assay. In both *P. infestans* and the other tested *Phytophthora* species, percentage inhibition was mainly driven by dose, which showed the largest effect size. This confirms a clear dose-dependent response, in agreement with previous studies reporting increasing inhibition with increasing EO concentration [27,30,39,40,42,44].

*Thymus serpyllum* EO showed stronger activity against *P. infestans* than *T. vulgaris* EO, reaching complete inhibition at 50 ppm (Table 3; Figure 4 and Figure 5). This result is relevant because *P. infestans* is one of the most destructive plant pathogens worldwide, and previous studies have already reported high sensitivity of this species to *Thymus* EOs [30,31,32,33,34,35,36]. The strong activity of TS-EO may be related to its carvacrol-rich profile, whereas TV-EO was characterized mainly by thymol, together with other compounds such as p-cymene and γ-terpinene. However, the observed differences should not be attributed exclusively to single major constituents, because interactions among compounds may strongly influence biological activity.

This interpretation is consistent with previous evidence suggesting that the biological activity of *Thymus* EOs depends on the entire chemical profile rather than solely on the dominant constituent. For example, Vettraino et al. [25] found that the antifungal efficacy of several *Thymus*-derived EOs against *Trametes versicolor*, *Gloeophyllum trabeum*, *Poria monticola*, and *Pleurotus ostreatus* was associated with the overall chemical profile rather than with the abundance of a single dominant compound. A key example was the comparison between *T. capitatus* and *C. capitatus* oils. Although *C. capitatus* contained a higher proportion of carvacrol, *T. capitatus* was more effective against *P. monticola* and *T. versicolor*. These findings suggest that compounds present in smaller amounts, such as p-cymene and γ-terpinene, may enhance or modulate the activity of the principal constituent through synergistic interactions.

For the other tested species, namely *P. palmivora*, *P. nicotianae*, *P. cactorum*, *P. cinnamomi*, *P. drechsleri*, and *P. citrophthora*, the multifactorial analysis showed significant effects of species, dose, and EO type, as well as significant interactions among these factors. These results indicate that inhibition depended not only on concentration and oil type, but also on the target *Phytophthora* species. This species-specific response is consistent with previous evidence showing that sensitivity to EOs can vary among pathogen species and isolates [32,35]. In the present study, all species showed a strong dose-dependent increase in inhibition, but the relative performance of TV-EO and TS-EO differed according to the pathogen and concentration. Such interactions are particularly important because they show that the efficacy of a given oil cannot be generalized across all *Phytophthora* species without specific testing.

The MIC and MFC data further supported the species-dependent activity of the two oils. TS-EO generally showed stronger inhibitory activity than TV-EO, as indicated by lower MIC values in some species. TS-EO was highly effective against *P. infestans*, with both MIC and MFC values of 50 ppm. TS-EO also showed a lower MIC than TV-EO against *P. citrophthora*, while both oils showed comparable activity against *P. palmivora* and *P. drechsleri*. These results suggest that TS-EO may have broader or stronger anti-*Phytophthora* activity under the tested conditions. Nevertheless, the response was not uniform across species, confirming that pathogen identity remains a key factor influencing EO efficacy.

Despite the promising inhibitory effects observed, these findings must still be interpreted within the limitations inherent to in vitro assays. Laboratory tests are essential for screening antimicrobial activity and comparing treatments under controlled conditions, but they cannot fully reproduce the complexity of plant–pathogen–environment interactions. EOs are also characterized by high volatility, low water solubility, chemical instability, and possible phytotoxicity, which may limit their direct application under practical conditions. Consequently, further greenhouse and field studies are needed to validate their disease-control potential, define suitable application strategies, and assess their safety on host plants. Such studies should also evaluate phytotoxicity, formulation stability, persistence, and delivery under biologically realistic conditions. In this context, formulation technologies, including nanoencapsulation, represent a promising approach to improve stability, persistence, controlled release, and delivery of EO bioactive compounds [27,54,55]. By improving protection, controlled release, and delivery of bioactive compounds, encapsulation systems may enhance EO persistence and efficacy, although their practical relevance must still be confirmed beyond in vitro conditions.

Currently, only sweet orange EO-based fungicides are registered in Italy [56]. However, the growing demand for new fungicidal solutions for pathogen control, particularly innovative formulations such as encapsulated bio-fungicides, is fully aligned with the European Union’s regulatory priorities. In fact, the EU has explicitly integrated the promotion of safer, biological plant-protection tools into its pesticide legislation, particularly through Directive 2009/128/EC on the sustainable use of pesticides.. This regulatory framework aims to encourage the development and uptake of new biopesticides, reflecting the Union’s broader strategy to reduce reliance on conventional chemical pesticides and expand the availability of effective biological alternatives. Nevertheless, the translation of *Thymus* EOs into practical plant-protection tools requires further experimental validation under realistic disease conditions.

## 5. Conclusions

In conclusion, *Thymus* EOs represent a promising source of bioactive compounds showing in vitro inhibitory activity against *Phytophthora* species, with efficacy that depends strongly on both EO chemotype and pathogen sensitivity. Within the scope of the focused literature surveyed in this review, the present findings indicate that these natural products may interfere with critical stages of pathogen development and therefore deserve further consideration as potential components of future sustainable crop protection strategies. However, the limited number of available studies and the narrow search strategy should be acknowledged, and the current evidence should therefore be interpreted as preliminary rather than definitive. Their practical exploitation, however, will depend on the integration of chemical standardization, advanced formulation technologies, and validation under biologically realistic conditions. In this perspective, the transition from laboratory evidence to effective field application will be the key step in determining the real agronomic value of *Thymus*-derived EOs.

## Figures and Tables

**Table 1:** Main chemical compounds (relative abundance) contained in TS-EO and TV-EO.

*Thymus serpyllum*		*Thymus vulgaris*	
Main Components	Contents (%)	Main Components	Contents (%)
			
carvacrol	68.49	thymol	47.9
linalool	8.22	p-cymene	15.8
p-cymene	5.24	γ-terpinene	10
γ-terpinene	4.02	carvacrol	4.4
thymol	2.59	linalool	4.1
ß-myrcene	1.42	β-caryophyllene	2.1
α-terpinene	1.19	β-myrcene	2
α-thujene	1.02	borneol	1.3
β-caryophyllene	0.94	α-terpinene	1.3
α-pinene	0.76	α-thujene	1.2
terpinen-4 ol	0.62	camphene	1.1
α-terpineol	0.61	α-bisabolene	1.1
c-sabinene hydrate	0.46	α-pinene	1.0
borneol	0.42	camphor	0.8
1.8 cineole	0.33	β-bisabolene	0.8
sabinene	0.28	eucalyptol	0.6
aromadendrene	0.27	carvacrol acetate	0.6
limonene	0.25	limonene	0.4
β-fellandrene	0.23	L-terpinen-4 ol	0.4
		α-terpineol	0.3
		β-pinene	0.2
		α-phellandrene	0.2
		trans-thujanol	0.2
		caryophyllene oxide	0.2
		cedrenol	0.2
		α-terpinolene	0.1

**Table 3:** Mycelial growth inhibition of TV-EO and TS-EO against *Phytophthora* species.

*Phytophthora* Species	Mycelial Growth Inhibition (%)							
	TV-EO				TS-EO			
	100 ppm	200 ppm	300 ppm	500 ppm	100 ppm	200 ppm	300 ppm	500 ppm
*P. cactorum* P69	41.02 ± 6.44 cB(A)	36.59 ± 8.48 cD(B)	61.03 ± 6.89 bB(B)	100.00 ± 0.00 aA(A)	7.56 ± 4.58 dAB(B)	77.61 ± 9.67 cC(A)	90.83 ± 3.04 bA(A)	100.00 ± 0.00 aA(A)
*P. cinnamomi* Ph28	−2.21 ± 14.36 cD(A)	77.71 ± 2.60 bB(B)	94.31 ± 2.15 aA(A)	100.00 ± 0.00 aA(A)	4.35 ± 16.06 cAB(A)	89.09 ± 2.27 bB(A)	96.08 ± 1.38 abA(A)	100.00 ± 0.00 aA(A)
*P. citrophthora* P6	0.73 ± 11.64 dD(A)	58.77 ± 8.07 cC(B)	89.84 ± 5.78 bA(B)	100.00 ± 0.00 aA(A)	1.76 ± 14.35 cB(A)	85.13 ± 11.28 bBC(A)	100.00 ± 0.00 aA(A)	100.00 ± 0.00 aA(A)
*P. drechsleri* P11	2.68 ± 10.18 bCD(A)	100.00 ± 0.00 aA(A)	100.00 ± 0.00 aA(A)	100.00 ± 0.00 aA(A)	2.51 ± 15.49 bAB(A)	100.00 ± 0.00 aA(A)	100.00 ± 0.00 aA(A)	100.00 ± 0.00 aA(A)
*P. nicotianae* Tu2.1	52.99 ± 16.52 cA(A)	85.45 ± 2.89 bB(B)	94.40 ± 0.00 aA(A)	94.40 ± 0.00 aA(A)	12.69 ± 6.02 bA(B)	94.40 ± 0.00 aAB(A)	93.28 ± 2.36 aA(A)	100.00 ± 0.00 aA(A)
*P. palmivora* P1	12.74 ± 18.44 bC(A)	4.65 ± 24.77 bE(B)	100.00 ± 0.00 aA(A)	100.00 ± 0.00 aA(A)	4.57 ± 18.13 bAB(A)	91.41 ± 4.36 aAB(A)	100.00 ± 0.00 aA(A)	100.00 ± 0.00 aA(A)
	12.5 ppm	25 ppm	37.5 ppm	50 ppm	12.5 ppm	25 ppm	37.5 ppm	50 ppm
*P. infestans* AM23	0.24 ± 7.05 d(B)	16.14 ± 10.66 c(B)	24.1 ± 11.43 b(B)	67.47 ± 16.95 a(B)	20.12 ± 8.05 c(A)	23.28 ± 8.26 c(A)	40.8 ± 5.05 b(A)	100 ± 0.00 a(A)

Values are expressed as mean ± standard deviation of mycelial growth inhibition percentage. Lowercase letters indicate significant differences among doses within the same species and EOs; uppercase letters indicate significant differences between *Phytophthora* species at the same dose and EOs; letters in brackets indicate significant differences between EOs within the same species and dose (ANOVA, *p* < 0.05).

**Table 4:** Minimum inhibitory concentration (MIC) and minimum fungicidal concentration (MFC) of *T. vulgaris* EO (TV-EO) and *T. serpyllum* EO (TS-EO) against the tested *Phytophthora* species. Values are reported in ppm for each species.

*Phytophthora* Species	TV-EO		TS-EO	
	MIC (ppm)	MFC (ppm)	MIC (ppm)	MFC (ppm)
*P. cactorum* P69	n.d.	n.d.	500	n.d.
*P. cinnamomi* Ph28	500	n.d.	500	n.d.
*P. citrophthora* P6	500	n.d.	300	500
*P. drechsleri* P11	200	500	200	500
*P. nicotianae* Tu2.1	n.d.	n.d.	500	n.d.
*P. palmivora* P1	300	500	300	500
*P. infestans* AM23	n.d.	n.d.	50	50

## Data Availability

The data presented in this study are available in the article and the Supplementary Materials.

## Supplementary Materials

The following supporting information can be downloaded at: https://www.mdpi.com/article/10.3390/pathogens15060582/s1, Table S1: PRISMA 2020 Checklist [57].
